# Biochemical and molecular investigation of thermal manipulation protocols during broiler embryogenesis and subsequent thermal challenge

**DOI:** 10.1186/s12917-015-0609-0

**Published:** 2015-12-01

**Authors:** Mohammad-Borhan Al-Zghoul, Sabry M. El-Bahr, Raida K. Al-Rukibat, Abd Elhafeed S. Dalab, Thnaian A. Althnaian, Saeed Y. Al-ramadan

**Affiliations:** Departments of Anatomy, College of Veterinary Medicine and Animal Resources, King Faisal University, Al-Ahsa, Saudi Arabia; Departments of Physiology, Biochemistry and Pharmacology (Biochemistry), College of Veterinary Medicine and Animal Resources, King Faisal University, Al-Ahsa, Saudi Arabia; Department of Biochemistry, Faculty of Veterinary Medicine, Alexandria University, Alexandria, Egypt; Department of Pathology and Animal Health, Faculty of Veterinary Medicine, Jordan University of Science and Technology, Irbid, Jordan; Department of Basic Medical Veterinary Sciences, Faculty of Veterinary Medicine, Jordan University of Science and Technology, P.O. Box 3030, Irbid, 22110 Jordan

**Keywords:** Thermal manipulation, Embryogenesis, Heat stress, Gene expression, Muscles, Plasma, Biochemistry

## Abstract

**Background:**

The aim of the current study was to evaluate the effect of different thermal manipulation (TM) protocols during embryogenesis on thermotolerance acquisition parameters during subsequent thermal challenge (TC) at posthatch day 28. A total of 1500 fertile chicken eggs were divided randomly into five treatments (300 eggs each): control was maintained at 37.8 °C and 56 % relative humidity (RH) whereas, TM_1,_ TM_2,_ TM_3_ and TM_4_ were subjected to 38.5, 39, 39.5 and 40 °C for 18 h and 65 % RH daily during embryonic days ED 12–18. Hatched chicks from each treatment group allocated randomly into two sub-treatment groups (thermo-neutral, naïve (TN) and thermal challenge (TC). At day 28 of age, chicks subjected to TC by adjusting room temperature to 42 °C for 6 h while naïve chicks kept under regular conditions (22 ± 1 °C and 50–60 % RH). Chick’s response to TC evaluated by determination of plasma T_3_, T_4_, corticosterone, total proteins, albumin, selected enzymes and some electrolytes at the beginning (0 h) and after 1, 3 and 5 h of TC in TM and TN chicks. Furthermore, pectoral and thigh muscles mRNA expression of Atrogin-1, CK, avUCP, DIO3, DIO2 were evaluated in TC and TN sub-treatment groups.

**Results:**

TM induced a significant reduction in free T_3_ and elevation in total proteins and albumin in plasma with significant down-regulation of Atrogin-1 and DIO2 and significant up-regulation of DIO3 mRNA expression in muscle of TM chicks compare to control. During TC at day 28, decrease in the concentrations of plasma free T_3_, total proteins and albumin with increase in T_4_ have been detected in control and TM chicks. TC induced up-regulation of Atrogin-1 and DIO3 with down-regulation of DIO2 gene expression in muscles of all TC chicks.

**Conclusion:**

The present study indicated that, TM improved thermotolerance acquisition by decreasing basal metabolic rate and muscle injury during thermal stress. Basal metabolic rate decreased via reduction of plasma T_3_ concentration with up and down regulation of expression of DIO3 and DIO2, respectively in muscles. Muscle injury protected by stimulation of protein biosynthesis and down-regulation of Atrogin-1 expression.

## Background

The detrimental effects of high ambient temperature on broiler performance have been extensively investigated [1, 2]. Several management protocols proposed to improve the thermotolerance acquisition in birds. Thermal manipulation (TM) at early and late stages of broiler embryogenesis is one of the most modern management model applied to improve the thermotolerance acquisition in broilers chickens [3–11]. TM using several temperatures (38.5, 39.0, 39.5 and 41.0 °C) for different durations (2, 3, 6, 12 and 24 h) during the incubation period has been investigated [4–11]. Blood biochemistry such as serum concentrations of glucose, ions, proteins, thyroid hormones, some enzyme activities and corticosterone considered as useful biomarkers of stress response [1, 12]. The ALT and CK activities increased in heat stressed chickens compare to control [13] whereas, serum total protein and albumin concentrations reduced in TM local chicks compare to control [14]. TM induced significant long-term effects on thyroid hormone metabolism by decrease the muscle mRNA expression of DIO3 and increase DIO2 mRNA expression in the liver [15]. Furthermore, a significant increase in muscle mRNA levels of Hsp70 during embryogenesis and during TC in posthatch chicks has been reported [3]. However, the effect of TM during embryogenesis and subsequent TC at posthatch day 28 on biochemical parameters and mRNA expression of stress related genes is not completely elucidated. In addition, the exact molecular and cellular bases for the improvement of the thermotolerance acquisition of the thermally manipulated chicks during thermal challenge are not fully understood.

Therefore, the objectives of the current study were to evaluate (a) the effect of TM at 38.5 °C, 39 °C, 39.5 °C and 40 °C for 18 h and 65 % RH daily during ED12–18 on selected biochemical parameters in plasma (free and bound thyroid hormones, total proteins, albumin, corticosterone, ALT, AST, ALP, CK, GGT, calcium, magnesium, sodium, potassium and chloride) and differential mRNA expression of Atrogin-1, CK, avUCP, DIO3, DIO2 in muscles of broiler chickens (b) a TM chick’s ability to cope with TC at posthatch day 28 as reflected on the above mentioned biochemical parameters when compared with control birds.

## Methods

### Incubating and hatching management

All experimental procedures and management conditions used in this study were approved by the King Faisal University Animal Care and Use Committee (KFU-ACUC). One thousand and seven hundred fertile Ross 315 broiler eggs obtained from one breeder flock (Riyadh, Saudi Arabia) were incubated in semi-commercial incubators (type OVA-Easy 380 Advance Series II, Brinsea, Sandford, UK). One thousand and five hundred normal eggs selected for an initial weight 62 ± 2 g. The selected eggs were then divided into five incubation treatment groups (300 each): control group was maintained at 37.8 °C 56 % relative humidity (RH) throughout the incubation period; TM_1_ was subjected to TM at 38.5 °C for 18 h and 65 % RH daily during ED12–18; TM_2_ was subjected to TM at 39 °C for 18 h and 65 % RH daily during ED12–18; TM_3_ was subjected to TM at 39.5 °C for 18 h and 65 % RH daily during ED12–18 and TM_4_ was subjected to TM at 40 °C for 18 h and 65 % RH daily during ED12–18. At hatching, chicks were distributed in floor pens at 32 °C and the temperature was gradually decreased to 21 °C at day 24 and maintained at 21 °C until day 35. Water and feed supplied to the chicks *ad libitum*. After feather drying, the one-day old chicks transferred to Veterinary Research Station, King Faisal University, Saudi Arabia for conduction of the field experiment.

### Thermal manipulation and heat challenge

Chicks from each treatment group divided randomly into two sub-treatment groups, Thermo-neutral (TN) and thermally challenged (TC). At post hatched day 28, forty chicks, randomly selected from the TC group were thermally challenged by adjusting room temperature to 42 °C for 6 h, respectively. TN chicks kept under regular condition (22 ± 1 °C and 50–60 % RH) in a separated room. At the beginning (0 h) and after 1, 3 and 5 h of TC, whole blood was drawn from the wing (brachial) vein from 5 chicks per treatment group and placed in an EDTA containing tubes. Identical samplings and measurements also conducted using the TN chicks as control. The harvested plasma were used for determination of free and bound thyroid hormones (T_3_, T_4_), corticosterone, selected enzyme activities (AST, ALT, ALP, CK, CGT), protein pattern (total protein and albumin) and electrolytes concentrations (Ca^+^, Mg^+^, Na^+^, Cl).

### Biochemical analysis of samples

Plasma was isolated by centrifugation of whole blood samples at 5,000 g for 10 min. Isolated plasma samples were then stored at −20 °C until further analysis. Commercial ELISA kits of Biocheck, Foster city, CA, USA were used for determination of total T_3_ (BC-1005), free T_3_ (BC-1006), total T_4_ (BC-1007), free T_4_ (BC-1008) and corticosterone on ELISA reader (Absorbance Microplate Reader ELx 800TM BioTek®, USA). The results calculated according to the manufacturer’s instruction. In addition, commercial kits of Reactvos biolab, Spain were used for determination of calcium, magnesium, potassium, sodium and chloride on EasyLyte Plus analyser (Medica corporation, Bedford, MA, USA). The commercial kits of Arab company for medical diagnostics (ARCOMEX), Jordan, were used for determination of the concentrations of total proteins and albumin and activities of ALP and CK whereas commercial diagnostic kits of Biosystem, Spain were used for estimation of ALT and AST activities on chemistry analyser and the results were calculated according to the manufacturer’s instruction.

### RNA isolation and semi-quantitative real time RT-PCR analyses

Pectoral and thigh muscles mRNA expressions of Atrogin1, Creatine kinase (CK), avian Uncoupling protein (avUCP), Deiodinase, iodothyronine, type III (DIO3), Deiodinase, iodothyronine, type II (DIO2) were evaluated using the semi-quantitative real time RT-PCR analyses. Pectoral and thigh muscles samples collected from 200 chicks (5 chicks from each treatment group per time point) at day 28 of age. Total RNA extracted from the muscle tissues, homogenated using the TRIzol/choloroform/isopropanol method followed by the removal of supernatants. The RNA pellet dissolved in diethylpyrocarbonate treated RNase free water (Ambion, Austin, TX, USA). DNA removed using DNase I kit (Ambion) and the RNA samples checked for concentration and purity (260:280 nm absorbency). RNA (2 μg) was reverse transcribed to cDNA in a reaction mixture using iScriptcDNA synthesis Kit BIO-RAD, Hercules, CA, USA). The cDNA used for semi-quantitative real-time RT-PCR using the CFX96 Touch™ Real time PCR (BIO-RAD). The semi-quantitative real time RT-PCR performed by the ssoAdvanced™ SYBR Green Supermix kit (BIO-RAD). Briefly, the 20 μl reaction mix was prepared from 10 μl of master mix; 2 μl forward primer (10 pmol); 2 μl reveres primer (10 pmol); 2 μl cDNA of the sample and 4 μl of nuclease-free water. Cycling parameters were 50 °C for 2 min, 95 °C for 15 min, 40 cycles of 95 °C for 10 s, followed by 30 s at 57 °C and 72 °C for 10 s with final melting at 95 °C for 20 s. Duplicates from each cDNA analyzed, fluorescence emission detected and relative quantification calculated automatically.

### Primers designing

Sequences of broiler Atrogin1, Creatine kinase (CK), Avian uncoupling protein (avUCP), deiodinase, iodothyronine, type III (DIO3), deiodinase, iodothyronine, type II (DIO2), and GAPDH were obtained from the gene bank (accession no: NM_00103956, NM_205507.1, AF433170.2, NM_001122648, NM_204114.3, NM_204305.1). Using these sequences, primers designed for each gene for the semi-quantitative real time RT-PCR analyses. All primers were designed using IDT Primer quest software (http://eu.idtdna.com/Primerquest/Home/Index). The sequences of the primers showed in Table 1.

**Table 1:** Primers for semi-quantitative real time RT-PCR analyses

Gene	Sequence (5′- 3′)
GAPDH	GTGTTATCATCTCAGCTCCCTCAG GGTCATAAGACCCTCCACAATG
Atrogin1	CGCAAACGGCTAATCCTATCT CTTCCGTGGGTAACACCTTATG
CK	TCTGGCACAACGACAACAA CCGGAAGACCTCCTTCATATTTC
avUCP	GCCCGCAACTCCATCATTA GGGACGTTGTCTGTCATCAG
DIO3	GCAGAAGCTGGACTTCTTCA CTGCCGAAGTTGAGGATGAG
DIO2	GGCTGACTTTCTGTTGGTCTAC CTTCCTGATTCCTGTGCTTCTT

### Statistical analysis

All statistical analyses performed using IBM SPSS statistics 21 software for Mac OS (IBM software, Chicago, USA). Data for the selected biochemical parameters, changes in control and TM treatment groups were analyzed by using One-way analysis of variance (ANOVA) followed by all-pairs Bonferroni test was used to compare different parameter and analysis of covariance (ANCOVA) for repeated measures, with the within-subject factors “treatment groups” and “time” (four time points (0, 1, 3 and 5 h of TC). Differences considered significant at *P* < 0.05.

## Results

The effects of TC (42 °C for 6 h; posthatch day 28) during 4 time points (at the beginning (0 h) and after 1, 3 and 5 h of TC on plasma free and bound thyroid hormones (T_3_ and T_4_) of broiler chicks subjected to different TM protocols at ED 12–18 showed in Table 2. The summarized data indicated that, a significant decrease (p <0.05) in free T_3_ concentration without changes in free T_4_ level has been reported in plasma of TM chicks compare to controls. The decrease of free T_3_ concentration in TM chicks does not influenced by changes in TM protocols (TM1-4). During TC at day 28, a significant decrease in plasma free T_3_ and increase in free T_4_ have been observed in control and TM chicks. Although, TC induced reduction in free T_3_ concentration of all TC chicks, this decrease was higher in TM birds compare to control. However, the concentrations of free T_4_ in plasma of TM birds were comparable to those of TN, control birds in spite of TC. TM and TC did not affect the concentrations of bound T_3_ and T_4_ throughout the experimental period.

**Table 2:** The effect of TC (42 °C for 6 h; posthatch day 28) during 4 time terminals (at the beginning and after 1, 3 and 5 h of TC) on plasma free and bound thyroid hormones (T_3_ and T_4_) of broiler chicks subjected to different TM protocols at ED 12–18 (*n* = 5)

Parameters	Groups	Treatments				
		Control	TM_1_	TM_2_	TM_3_	TM_4_
Free T_3_	TN	3.63 ± 0.16 ^aw^	2.66 ± 0.29 ^bw^	2.70 ± 0.11 ^bw^	2.65 ± 0.13 ^bw^	2.60 ± 0.16 ^bw^
	TC_1_	2.43 ± 0.28 ^ax^	1.33 ± 0.16 ^bx^	1.32 ± 0.29 ^bx^	1.79 ± 0.25 ^bx^	1.65 ± 0.28 ^bx^
	TC_3_	2.47 ± 0.20 ^ax^	1.47 ± 0.14 ^bx^	1.40 ± 0.31 ^bx^	1.79 ± 0.15 ^bx^	1.67 ± 0.16 ^bx^
	TC_5_	2.81 ± 0.30 ^ax^	1.41 ± 0.14 ^bx^	1.42 ± 0.27 ^bx^	1.72 ± 0.12 ^bx^	1.81 ± 0.23 ^bx^
Bound T_3_	TN	4.05 ± 0.01 ^aw^	3.90 ± 0.29 ^aw^	3.75 ± 0.35 ^aw^	3.68 ± 0.54 ^aw^	3.34 ± 0.37 ^aw^
	TC_1_	3.57 ± 0.51 ^aw^	3.68 ± 0.78 ^aw^	3.57 ± 0.23 ^aw^	3.79 ± 0.24 ^aw^	3.54 ± 0.28 ^aw^
	TC_3_	3.50 ± 0.53 ^aw^	3.62 ± 0.78 ^aw^	3.96 ± 0.03 ^aw^	3.96 ± 0.59 ^aw^	3.65 ± 0.60 ^aw^
	TC_5_	3.94 ± 0.40 ^aw^	3.89 ± 0.89 ^aw^	3.76 ± 0.07 ^aw^	3.79 ± 0.33 ^aw^	3.67 ± 0.91 ^aw^
Free T_4_	TN	0.21 ± 0.12 ^aw^	0.29 ± 0.14 ^aw^	0.34 ± 0.12 ^aw^	0.30 ± 0.13 ^aw^	0.38 ± 0.18 ^aw^
	TC_1_	0.69 ± 0.10 ^ax^	0.69 ± 0.16 ^ax^	0.40 ± 0.13 ^ax^	0.50 ± 0.12 ^ax^	0.51 ± 0.13 ^ax^
	TC_3_	0.52 ± 0.13 ^ax^	0.58 ± 0.14 ^ax^	0.59 ± 0.21 ^ax^	0.58 ± 0.17 ^ax^	0.56 ± 0.11 ^ax^
	TC_5_	0.54 ± 0.12 ^ax^	0.51 ± 0.13 ^ax^	0.56 ± 0.11 ^ax^	0.56 ± 0.11 ^ax^	0.55 ± 0.11 ^ax^
Bound T_4_	TN	2.56 ± 0.13 ^aw^	2.19 ± 0.45 ^aw^	2.87 ± 0.61 ^aw^	2.65 ± 0.42 ^aw^	2.82 ± 0.60 ^aw^
	TC_1_	2.56 ± 0.46 ^aw^	2.88 ± 0.17 ^aw^	2.67 ± 0.32 ^aw^	2.86 ± 0.55 ^aw^	2.62 ± 0.20 ^aw^
	TC_3_	2.02 ± 0.57 ^aw^	2.71 ± 0.47 ^aw^	2.71 ± 0.23 ^aw^	2.33 ± 0.51 ^aw^	2.78 ± 0.25 ^aw^
	TC_5_	2.98 ± 0.54 ^aw^	2.30 ± 0.34 ^aw^	2.94 ± 0.16 ^aw^	2.95 ± 0.01 ^aw^	2.45 ± 0.41 ^aw^

*Control* 37.8 °C, *TM*_*1*_ Thermal manipulation at 38.5 °C for 18 h, *TM*_*2*_ Thermal manipulation at 39 °C for 18 h, *TM*_*3*_ Thermal manipulation at 39.5 °C for 18 h, *TM*_*4*_ Thermal manipulation at 40 °C for 18 h, *TC* Thermal challenged

^a–b^ within rows, means ± SD with different superscripts differ significantly (*P* < 0.05)

^w-x^ Between naïve and TC chicks within a parameter, means ± SD with different superscripts differ significantly (*P* < 0.05)

The data of Table 3 showed the effect of TC (42 °C for 6 h; posthatch day 28) during 4 time points (at the beginning and after 1, 3 and 5 h of TC) on total proteins, albumin and corticosterone of broiler chicks subjected to different TM protocols at ED 12–18. These findings indicated that, TM induced a significant increase in plasma total proteins and albumin of TM chicks compared to TN chicks, control. The increase of total proteins and albumin values in TM chicks does not influenced by changes in TM protocols (TM1-4). TC at day 28 induced significant reduction in total proteins and albumin in TM and control chicks. Although, TC induced reduction in total proteins and albumin concentrations of all TC chicks, this decrease was higher in control birds than TM birds. TM and TC did not affect the concentration of corticosterone throughout the experimental period (Table 3).

**Table 3:** The effect of TC (42 °C for 6 h; posthatch day 28) during 4 time terminals (at the beginning and after 1, 3 and 5 h of TC) on plasma protein pattern and cortisol of broiler chicks subjected to different TM protocols at ED 12–18 (*n* = 5)

Parameters	Groups	Treatments				
		Control	TM_1_	TM_2_	TM_3_	TM_4_
Total proteins	TN	28.4 ± 0.3 ^aw^	37.5 ± 1.1 ^bw^	36.0 ± 1.3 ^bw^	35.0 ± 1.4 ^bw^	37.1 ± 1.4 ^bw^
	TC_1_	21.9 ± 0.1 ^ax^	27.2 ± 1.0 ^bx^	27.5 ± 0.9 ^bx^	26.6 ± 1.1 ^bx^	26.8 ± 1.6 ^bx^
	TC_3_	22.7 ± 0.2 ^ax^	27.4 ± 1.1 ^bx^	27.5 ± 1.1 ^bx^	26.7 ± 1.0 ^bx^	27.5 ± 1.2 ^bx^
	TC_5_	22.6 ± 1.5 ^ax^	29.6 ± 0.6 ^bx^	28.0 ± 1.0 ^bx^	28.1 ± 1.0 ^bx^	28.2 ± 0.8 ^bx^
Albumin	TN	17.5 ± 0.6 ^aw^	20.2 ± 1.1 ^bw^	20.2 ± 1.0 ^bw^	20.3 ± 1.2 ^bw^	20.3 ± 1.1 ^bw^
	TC_1_	12.0 ± 0.2 ^ax^	15.5 ± 0.5 ^bx^	15.4 ± 0.3 ^bx^	14.0 ± 0.7 ^bx^	14.8 ± 0.4 ^bx^
	TC_3_	12.1 ± 0.2 ^ax^	15.2 ± 0.8 ^bx^	15.9 ± 0.7 ^bx^	14.7 ± 1.0 ^bx^	14.9 ± 1.0 ^bx^
	TC_5_	12.4 ± 0.1 ^ax^	15.1 ± 0.7 ^bx^	14.7 ± 0.4 ^bx^	14.6 ± 1.7 ^bx^	15.1 ± 2.3 ^bx^
Corticosterone	TN	0.21 ± 0.03^aw^	0.21 ± 0.02 ^aw^	0.22 ± 0.01 ^aw^	0.22 ± 0.03 ^aw^	0.20 ± 0.02 ^aw^
	TC_1_	0.21 ± 0.00 ^aw^	0.20 ± 0.01 ^aw^	0.21 ± 0.01 ^aw^	0.22 ± 0.05 ^aw^	0.21 ± 0.03 ^aw^
	TC_3_	0.21 ± 0.01 ^aw^	0.21 ± 0.11 ^aw^	0.21 ± 0.01 ^aw^	0.21 ± 0.12 ^aw^	0.21 ± 0.01 ^aw^
	TC_5_	0.22 ± 0.05 ^aw^	0.21 ± 0.05 ^aw^	0.20 ± 0.01 ^aw^	0.21 ± 0.05 ^aw^	0.22 ± 0.01 ^aw^

*Control* 37.8 °C, *TM*_*1*_ Thermal manipulation at 38.5 °C for 18 h, *TM*_*2*_ Thermal manipulation at 39 °C for 18 h, *TM*_*3*_ Thermal manipulation at 39.5 °C for 18 h, *TM*_*4*_ Thermal manipulation at 40 °C for 18 h, *TC* Thermal challenged

^a–b^ within rows, means ± SD with different superscripts differ significantly (*P* < 0.05)

^w-x^ Between naïve and TC chicks within a parameter, means ± SD with different superscripts differ significantly (*P* < 0.05)

The effect of TC (42 °C for 6 h; posthatch day 28) during 4 time points (at the beginning and after 1, 3 and 5 h of TC) on plasma ALT, AST, GGT and CK activities of broiler chicks subjected to different TM protocols at ED 12–18 is summarized in Table 4. The data indicated that, neither TM nor TC at day 28 resulted in changes in the activities of these enzymes throughout the experimental period (Table 4).

**Table 4:** The effect of TC (42 °C for 6 h; posthatch day 28) during 4 time terminals (at the beginning and after 1, 3 and 5 h of TC) on selected enzymes activities of broiler chicks subjected to different TM protocols at ED 12–18 (*n* = 5)

Parameters	Groups	Treatments				
		Control	TM_1_	TM_2_	TM_3_	TM_4_
ALT	TN	5.5 ± 1.7 ^aw^	6.5 ± 1.9 ^aw^	5.5 ± 0.7 ^aw^	7.0 ± 1.4 ^aw^	6.5 ± 0.7 ^aw^
	TC_1_	6.0 ± 2.4 ^aw^	8.5 ± 2.1 ^aw^	8.0 ± 1.9 ^aw^	9.5 ± 1.5 ^aw^	8.5 ± 1.5 ^aw^
	TC_3_	7.5 ± 0.4 ^aw^	9.0 ± 1.7 ^aw^	7.5 ± 0.7 ^aw^	8.5 ± 0.7 ^aw^	8.5 ± 1.6 ^aw^
	TC_5_	5.5 ± 2.1 ^aw^	6.0 ± 1.6 ^aw^	8.5 ± 1.5 ^aw^	9.0 ± 1.6 ^aw^	9.5 ± 1.7 ^aw^
AST	TN	80.5 ± 0.7 ^aw^	75.8 ± 4.1 ^aw^	76.5 ± 3.5 ^aw^	83.5 ± 2.3 ^aw^	83.5 ± 6.4 ^aw^
	TC_1_	80.5 ± 6.4 ^aw^	76.5 ± 7.6 ^aw^	80.0 ± 1.4 ^aw^	83.5 ± 2.0 ^aw^	81.0 ± 8.5 ^aw^
	TC_3_	85.0 ± 2.8 ^aw^	70.0 ± 1.2 ^aw^	77.0 ± 1.4 ^aw^	72.0 ± 2.9 ^aw^	76.0 ± 4.2 ^aw^
	TC_5_	87.5 ± 7.7 ^aw^	80.5 ± 4.9 ^aw^	77.5 ± 6.4 ^aw^	70.0 ± 3.2 ^aw^	80.5 ± 3.5 ^aw^
GGT	TN	10.5 ± 3.5 ^aw^	11.5 ± 0.7 ^aw^	11.5 ± 0.7 ^aw^	12.5 ± 0.7 ^aw^	11.0 ± 1.4 ^aw^
	TC_1_	11.5 ± 0.7 ^aw^	11.5 ± 0.7 ^aw^	11.8 ± 0.6 ^aw^	10.5 ± 2.1 ^aw^	12.5 ± 0.7 ^aw^
	TC_3_	11.5 ± 1.0 ^aw^	12.0 ± 0.7 ^aw^	12.5 ± 0.7 ^aw^	12.0 ± 1.4 ^aw^	12.5 ± 0.7 ^dw^
	TC_5_	11.5 ± 0.9 ^aw^	9.5 ± 2.1 ^aw^	12.5 ± 0.5 ^aw^	10.0 ± 1.2 ^aw^	11.0 ± 0 .9 ^aw^
CK	TN	1276 ± 79^aw^	1277 ± 100 ^aw^	1394 ± 98 ^aw^	1239 ± 99 ^aw^	1293 ± 141 ^aw^
	TC_1_	1202 ± 80 ^aw^	1314 ± 108 ^aw^	1182 ± 150 ^aw^	1135 ± 161 ^aw^	1205 ± 138 ^aw^
	TC_3_	1310 ± 100 ^aw^	1410 ± 100 ^aw^	1253 ± 138 ^aw^	1239 ± 163 ^aw^	1285 ± 124 ^aw^
	TC_5_	1239 ± 95 ^aw^	1307 ± 150 ^aw^	1257 ± 96 ^aw^	1205 ± 144 ^aw^	1265 ± 90 ^aw^

*Control* 37.8 °C, *TM*_*1*_ Thermal manipulation at 38.5 °C for 18 h, *TM*_*2*_ Thermal manipulation at 39 °C for 18 h, *TM*_*3*_ Thermal manipulation at 39.5 °C for 18 h, *TM*_*4*_ Thermal manipulation at 40 °C for 18 h, *TC* Thermal challenged

^a–d^ within rows, means ± SD with different superscripts differ significantly (*P* < 0.05)

^w-z^ Between naïve and TC chicks within a parameter, means ± SD with different superscripts differ significantly (*P* < 0.05)

The data of Table 5 showed the effect of TC (42 °C for 6 h; posthatch day 28) during 4 time points (at the beginning and after 1, 3 and 5 h of TC) on the concentrations of calcium, magnesium, sodium, potassium and chloride of broiler chicks subjected to different TM protocols at ED 12–18. This data indicated that, during TC at day 28, the concentrations of these elements were comparable in all treatments including the control. Furthermore, TM did not affect the concentrations of these elements in all treatments.

**Table 5:** The effect of TC (42 °C for 6 h; posthatch day 28) during 4 time terminals (at the beginning and after 1, 3 and 5 h of TC) on selected plasma electrolytes concentrations of broiler chicks subjected to different TM protocols at ED 12–18 (*n* = 5)

Parameters	Groups	Treatments				
		Control	TM_1_	TM_2_	TM_3_	TM_4_
Calcium	TN	13.5 ± 0.7 ^aw^	13.0 ± 1.4 ^aw^	12.8 ± 0.3 ^aw^	12.5 ± 0.7 ^aw^	11.9 ± 2.6 ^aw^
	TC_1_	12.6 ± 0.6 ^aw^	12.9 ± 1.8 ^aw^	12.7 ± 0.5 ^aw^	11.5 ± 3.3 ^aw^	12.0 ± 0.0 ^aw^
	TC_3_	13.8 ± 1.2 ^aw^	13.0 ± 1.4 ^aw^	13.5 ± 0.7 ^aw^	11.9 ± 1.8 ^aw^	12.4 ± 0.5 ^aw^
	TC_5_	12.5 ± 0.8 ^aw^	12.0 ± 1.2 ^aw^	13.5 ± 3.0 ^aw^	12.8 ± 0.8 ^aw^	13.0 ± 2.2 ^aw^
Magnesium	TN	4.1 ± 1.4 ^aw^	4.0 ± 1.1 ^aw^	3.8 ± 1.5 ^aw^	4.9 ± 1.6 ^aw^	4.2 ± 1.6 ^aw^
	TC_1_	3.9 ± 1.1 ^aw^	3.5 ± 1.3 ^aw^	4.2 ± 1.4 ^aw^	3.2 ± 1.5 ^aw^	3.9 ± 1.1 ^aw^
	TC_3_	3.7 ± 1.3 ^aw^	4.1 ± 1.2 ^aw^	3.7 ± 1.1 ^aw^	3.7 ± 1.3 ^aw^	3.2 ± 1.6 ^aw^
	TC_5_	4.0 ± 1.1 ^aw^	4.6 ± 1.8 ^aw^	4.0 ± 1.6 ^aw^	4.7 ± 1.1 ^aw^	5.4 ± 1.8 ^aw^
Sodium	TN	146.3 ± 5.9 ^aw^	147.9 ± 1.6 ^aw^	142.5 ± 3.5 ^aw^	142.4 ± 0.8 ^aw^	146.5 ± 6.4 ^aw^
	TC_1_	142.5 ± 3.5 ^aw^	137.7 ± 1.7 ^aw^	139.3 ± 2.0 ^aw^	138.5 ± 2.1 ^aw^	142.5 ± 5.8 ^aw^
	TC_3_	139.6 ± 5.2 ^aw^	140.7 ± 2.1 ^aw^	141.5 ± 0.7 ^aw^	141.7 ± 6.6 ^aw^	140.0 ± 4.2 ^aw^
	TC_5_	138.0 ± 6.9 ^aw^	143.6 ± 3.7 ^aw^	138.1 ± 5.0 ^aw^	142.2 ± 9.5 ^aw^	143.6 ± 3.6 ^aw^
Potassium	TN	9.7 ± 1.3 ^aw^	10.4 ± 1.4 ^aw^	10.5 ± 0.2 ^aw^	9.8 ± 1.3 ^aw^	9.4 ± 1.4 ^aw^
	TC_1_	10.3 ± 1.5 ^aw^	9.4 ± 1.1 ^aw^	10.9 ± 1.0 ^aw^	9.4 ± 1.6 ^aw^	9.8 ± 1.4 ^aw^
	TC_3_	9.5 ± 1.6 ^aw^	10.7 ± 1.3 ^aw^	10.3 ± 1.2 ^aw^	9.7 ± 1.1 ^aw^	11.7 ± 1.4 ^aw^
	TC_5_	9.2 ± 1.3 ^aw^	9.8 ± 1.3 ^aw^	8.8 ± 2.6 ^aw^	8.8 ± 1.4 ^aw^	9.9 ± 1.8 ^aw^
Chloride	TN	113.0 ± 1.8 ^aw^	112.5 ± 2.1 ^aw^	114.0 ± 1.1 ^aw^	110.5 ± 2.9 ^aw^	107.5 ± 7.5 ^aw^
	TC_1_	116.5 ± 3.7 ^aw^	109.5 ± 4.8 ^aw^	116.0 ± 1.4 ^aw^	113.0 ± 1.8 ^aw^	112.0 ± 3.1 ^aw^
	TC_3_	118.5 ± 3.9 ^aw^	116.0 ± 1.4 ^aw^	116.0 ± 1.4 ^aw^	115.5 ± 2.1 ^aw^	114.5 ± 2.1 ^aw^
	TC_5_	114.5 ± 0.5 ^aw^	112.0 ± 0.9 ^aw^	112.0 ± 0.7 ^aw^	114.5 ± 3.5 ^aw^	115.0 ± 1.4 ^aw^

*Control* 37.8 °C, *TM*_*1*_ Thermal manipulation at 38.5 °C for 18 h, *TM*_*2*_ Thermal manipulation at 39 °C for 18 h, *TM*_*3*_ Thermal manipulation at 39.5 °C for 18 h, *TM*_*4*_ Thermal manipulation at 40 °C for 18 h, *TC* Thermal challenged

^a–d^ within rows, means ± SD with different superscripts differ significantly (*P* < 0.05)

^w-z^ Between naïve and TC chicks within a parameter, means ± SD with different superscripts differ significantly (*P* < 0.05)

The effect of TC (42 °C for 6 h; posthatch day 28) during 4 time terminals (at the beginning and after 1, 3 and 5 h of TC) on relative mRNA expression of Atrogin-1, cKM, avUCP, DIO3 and DIO2 in the muscles of broiler chicks subjected to different TM protocols at ED 12–18 are shown in Table 6. This data indicated that, TM induced a significant down-regulation of Atrogin-1 and DIO2 and significant up-regulation of gene expression of DIO3 in muscle of TM chicks compared to TN chicks, control without any significant changes among TM protocols (TM1-4). TC induced up-regulation of gene expression of Atrogin-1 and DIO3 and down-regulation of gene expression of DIO2 in control and TM chicks. Although, TC induced up-regulation of Atrogin-1 and DIO3 and down-regulation of DIO2 expression in muscle of all TC chicks, the up-regulation of Atrogin-1 and DIO3 and down-regulation of DIO2 expression in muscle of TM birds were higher than that of control. TM and TC did not affect the gene expression of CKM and avUCP in muscles of investigated chicks.

**Table 6:** The effect of TC (42 °C for 6 h; posthatch day 28) during 4 time terminals (at the beginning and after 1, 3 and 5 h of TC) on relative mRNA levels of Atrogin-1, cKM, avUCP, DIO3 and DIO2 in the Muscle of broiler chicks subjected to different TM protocols at ED 12–18 (*n* = 5)

Parameters	Groups	Treatments				
		Control	TM_1_	TM_2_	TM_3_	TM_4_
Atrogin-1	TN	1.00 ± 0.05^aw^	0.20 ± 0.01^bw^	0.26 ± 0.05^bw^	0.28 ± 0.08^bw^	0.30 ± 0.09^bw^
	TC_1_	10.65 ± 0.02^ax^	5.60 ± 1.06^bx^	6.40 ± 1.14^bx^	5.52 ± 1.04^bx^	6.61 ± 1.05^bx^
	TC_3_	20.13 ± 0.82^ay^	10.99 ± 1.50^by^	9.52 ± 1.08^by^	9.11 ± 1.88^by^	9.66 ± 1.44^by^
	TC_5_	28.30 ± 5.40 ^az^	16.02 ± 1.10^bz^	15.21 ± 1.34^bz^	16.11 ± 1.40^bz^	15.96 ± 1.21^bz^
cKM	TN	1.00 ± 0.04 ^aw^	1.29 ± 0.03 ^aw^	1.25 ± 0.03 ^aw^	1.25 ± 0.03 ^aw^	1.36 ± 0.02 ^aw^
	TC_1_	1.60 ± 0.52 ^ax^	1.33 ± 0.33 ^aw^	1.95 ± 0.81 ^ax^	1.95 ± 0.88 ^ax^	1.66 ± 0.42 ^aw^
	TC_3_	1.96 ± 0.81 ^ax^	1.56 ± 0.43 ^aw^	1.55 ± 0.32 ^aw^	1.72 ± 0.66 ^aw^	1.53 ± 0.51 ^aw^
	TC_5_	1.89 ± 0.84 ^ax^	1.04 ± 0.22 ^aw^	1.37 ± 0.63 ^aw^	1.44 ± 0.32 ^aw^	1.45 ± 0.40 ^aw^
avUCP	TN	1.00 ± 0.32 ^aw^	0.97 ± 0.71 ^aw^	1.34 ± 0.24 ^aw^	1.43 ± 0.40 ^aw^	1.39 ± 0.19 ^aw^
	TC_1_	1.30 ± 0.21 ^aw^	1.18 ± 0.31 ^aw^	1.20 ± 0.11 ^aw^	1.70 ± 0.51 ^aw^	1.05 ± 0.21 ^aw^
	TC_3_	1.87 ± 0.60 ^ax^	1.30 ± 0.29 ^aw^	1.08 ± 0.33 ^aw^	1.37 ± 0.23 ^aw^	1.33 ± 0.21 ^aw^
	TC_5_	1.63 ± 0.40 ^aw^	1.10 ± 0.24 ^aw^	1.54 ± 0.45 ^aw^	1.09 ± 0.21 ^aw^	1.79 ± 0.71 ^aw^
DIO3	TN	2.00 ± 0.01^aw^	2.36 ± 0.01^bw^	2.69 ± 0.01^bw^	2.63 ± 0.03^bw^	2.78 ± 0.03^bw^
	TC_1_	3.43 ± 0.07^ax^	4.43 ± 0.02 ^bx^	4.25 ± 0.08 ^bx^	4.27 ± 0.03 ^bx^	4.29 ± 0.08 ^bx^
	TC_3_	3.42 ± 0.06^ax^	4.37 ± 0.10 ^bx^	4.34 ± 0.03 ^bx^	4.32 ± 0.05 ^bx^	4.31 ± 0.02 ^bx^
	TC_5_	3.56 ± 0.06^ax^	4.32 ± 0.03 ^bx^	4.38 ± 0.07 ^bx^	4.34 ± 0.01 ^bx^	4.37 ± 0.08 ^bx^
DIO2	TN	4.88 ± 0.01^aw^	2.27 ± 0.01^bw^	2.27 ± 0.03^bw^	2.26 ± 0.02^bw^	2.25 ± 0.03^bw^
	TC_1_	3.17 ± 0.03^ax^	1.87 ± 0.08^bx^	1.90 ± 0.03^bx^	1.89 ± 0.01^bx^	1.13 ± 0.03^bx^
	TC_3_	3.25 ± 0.02^ax^	1.90 ± 0.02^bx^	1.13 ± 0.08^bx^	1.32 ± 0.09^bx^	1.34 ± 0.04^bx^
	TC_5_	3.15 ± 0.03^ax^	1.86 ± 0.01^bx^	1.32 ± 0.03^bx^	1.34 ± 0.04^bx^	1.38 ± 0.04^bx^

^a-w^Within the same row, means ± SD with different superscripts differ significantly (*P* <0.05)

^w-z^Within the same gene, means ± SD with different superscripts differ significantly (*P* <0.05)

## Discussion

The importance of thyroid gland hormones in adaptation to heat stress is related to the central role that thyroid hormones play in the regulation of metabolic rate of birds [16]. This effect has been demonstrated by thyroid hormone administration, which stimulates heat production [17] and by surgical or chemical thyroidectomy of chicken, which produces a decrease in metabolic rate and body temperature [18]. The current findings indicated that TM during embryogenesis was able to affect the thermoregulatory events of broiler chickens, which resulted in a lowered free T_3_ in TM chicks compared with control. In addition, TC at posthatch day 28 reduced free T_3_ in TM and control birds. It is well known that, the epigenetic heat adaptation involves changes in blood contents, hormonal and metabolic reaction that enhances heat endurance [14]. Parallel to the current findings, the concentration of T_3_ reduced in heat acclimation treated than control broilers [19]. In consistent with the present study, TC (41°C/6 h on days 3, 7 and 42 of age) caused a reduction in T_3_ level in TC chicks compared with TN chicks [3]. The current finding indicated that TC at posthatch day 28 increased T_4_ level in TC chicks compared with TN chicks. This finding come in accordance with the previous results [3] which demonstrated that, TC (41°C/6 h on days 3, 7 and 42 of age) caused an increase in T_4_ level in TM chicks compared with TN chicks. The current findings also indicate that, TM at ED (12–18) induced significant reduction in T_3_ level without changes in T_4_ level in TM birds compare to control. This finding agrees with previous report [3] with regard to T_3_.

TM resulted in significant increases in plasma total proteins and albumin of TM chicks compared to control chicks. Although, TC at posthatch day 28 induced a significant reduction of both plasma total proteins and albumin concentrations in TM and control birds, the concentrations of these proteins in TM birds were still higher than that of control. This evidenced that, TM modulates the thermoregulation process during embryogenesis and posthatching stages. Previously, significant reduction in plasma total protein and albumin was observed when embryos were exposed to high incubation temperature than the control [20]. In addition, it is well known that, heat stress leads to delay the synthesis of most proteins except heat shock proteins [21]. Serum total protein and albumin concentrations were significantly decreased in one day hatched local Egyptian chicks subjected to TM (40 °C; RH 55 %; ED 14–17) compared to control [14]. As reported in the current study, the increment of total proteins and albumin concentrations in TM birds can considered as a sort of protection of muscle mass against injury induced by TC.

Publications about the effect of heat stress on enzymes activities are contradictory, possibly due to the large variability of the enzymes [22, 23]. The current findings indicated that, TM did not change the activities of examined enzymes activities throughout the experimental period. Similarly, subsequent TC at posthatch day 28 did not affect the enzyme activities of TM and control birds. According to the obtained results, heat exposure did not significantly change the enzyme activities in the chicken’s serum. This is supported by previous finding reported that, the activities of CK and AST were not influenced even by acute heat exposure [24]. The current findings suggest that virtually no cellular damage, resulting in leakage of intracellular enzymes into the blood, took place. Therefore, the thermtolerance acquisition induced by TM was safe to the birds’ tissues.

Publications concerning the effect of high ambient temperature on serum electrolytes status in chickens are contradicting. Although, non-significant changes of all examined electrolytes have been observed in plasma of TM and subsequent TC at posthatch day 28 chicks in the current study, Khone et al. [25] reported increased blood potassium level in heat stress birds whereas, Ait-Boulahsen et al. [26] noted otherwise. Lin et al. [12] reported that, chloride level was increased and potassium level was decreased by high temperature in birds. McDaniel et al. [27, 28] reported a significant decrease of calcium and phosphorus levels in heat stressed birds. Furthermore, Zulkifli et al. [29] reported that serum levels of sodium and chloride were significantly higher and serum potassium were significantly lower 14 days after heat exposure in broiler chickens.

DIO2 acts as an outer-ring 5ʹ-deiodinase by removing iodine from the 5ʹ outer-ring site to convert T_4_ to the more active form T_3_, while DIO3 acts as an inner-ring deiodinase 5-deiodinase to remove iodine from the inner-ring of T_4_ and T_3_ and convert these hormones to inactive forms including reverse triiodothyronine (rT_3_) and diiodothyronine (T_2_) [15]. The current data indicated that, TM induced a significant down-regulation of DIO2 and significant up-regulation of gene expression of DIO3 in muscle of TM chicks compared to control. Furthermore, TC at posthatch day 28 induced up-regulation of DIO3 and down-regulation of DIO2 gene expression in TM chicks and in control birds however, this effect was higher in TM birds compare to control. The lower DIO2 and higher DIO3 expression in TM birds is consistent with the decrease in plasma T_3_ concentration reported in the current study and also with decrease of plasma T_3_ reported previously in birds and mammals [30, 31] and fast growing chickens [15]. The persistence of increase of DIO3 and decrease of DIO2 expression in TM birds than control during TC might explain why plasma T_3_ concentrations in TM birds were lower than that of control during TC. Nevertheless, TM decreased O_2_ consumption of embryos, indicating a potentially lower metabolic rate in these animals [5, 32]. The current study showed that, mRNA expression of DIO3 significantly increased while that of DIO2 significantly decreased in muscle of TM birds. These findings suggest that TM has a long-term overall negative effect on thyroid hormone metabolism in muscle. However, the physiological impact of such changes remains to be determined, since the activity of deiodinase enzymes is not necessarily correlated with their mRNA expression.

Atrogin-1 an E3 ubiquitin ligase also referred to as MAFbx (muscle atrophy F-box), plays a vital role in muscle atrophy [33, 34]. Atrogin-1 expressed only in skeletal muscle. Its expression increased in catabolic conditions that result in muscle atrophy [34, 35]. The current results indicated that, TM induced a significant down-regulation of Atrogin-1 compared to the control. Moreover, TC at posthatch day 28 induced up-regulation of gene expression of this gene in muscle of TM and control chicks. However, the up-regulation of Atrogin-1 expression in muscle of TM birds was lower than that of control. These findings indicated that, all tested TM protocols decrease the effect of muscle degeneration and this is consistent with non-significant changes that observed in enzymes activities of CK. In response to TC, Atrogin-1 was up regulated and this dynamic profile is consistent with previous reports of muscle injury response, whereby the expression of atrogin-1 was concomitant with atrophy and recovery [36]. Atrogin-1 regulated by FOXO transcription factors, via nuclear localization of FOXO in response to various stimuli that inhibit PI3K/Akt (protein Kinase B) signaling and subsequently drive atrophy [37, 38]. The mechanism of down-regulation of Atrogin-1 by TM could be furtherly investigated and studying the phosphorylation of Akt/FOXO axis might be of great benefits. As reported above, the increment of plasma total proteins and albumin concentrations together with down-regulation of Atrogin-1 in muscle of TM birds are two mechanisms that perhaps induced by TM as a sort of protection of muscle mass against possible atrophy induced by TC. The current study demonstrated that TM and subsequent TC did not affect the gene expression of cKM and avUCP in muscles of all investigated chicks. The current findings regarding the cKM are in consistent with the non-significant changes that observed in serum CK enzyme activities of all TC chicks. In addition, the down-regulation of Atrogin-1 and elevation of total proteins confirmed these findings. The non-significant changes of avUCP in muscle of all TC birds might give an indication that, the action taken by the body of birds subjected to TM was enough to counter act the thermal challenges.

In conclusion, the present study indicated that, TM improved thermotolerance acquisition by decreasing each of basal metabolic rate and muscle atrophy. The decrease in basal metabolic rate induced via reduction of T_3_ concentration in plasma with up and down regulation of DIO3 and DIO2, respectively. However, the decrease in muscle atrophy conducted by stimulation of protein biosynthesis and down-regulation of Atrogin-1 gene expression.

## Conclusion

In conclusion, our findings strongly indicated that, TM improved thermotolerance acquisition by decreasing basal metabolic rate and muscle injury during thermal stress. Basal metabolic rate decreased via reduction of plasma T_3_ concentration with up and down regulation of expression of DIO3 and DIO2, respectively in muscles. Muscle injury protected by stimulation of protein biosynthesis and down-regulation of Atrogin-1 expression.

## Abbreviations

TM: Thermal manipulation
TC: Thermal challenge
TN: Thermal-neutral naïve
RH: Relative humidity
ED: Embryonic day
avUCP: Avian uncoupling protein
DIO3: Deiodinase, iodothyronine, type III
DIO2: Deiodinase, iodothyronine, type II
AST: Aspartate transaminase
ALT: Alanine transaminase
ALP: Alkaline phosphatase
CK: Creatine kinase
CGT: Gamma-glutamyl transferase
T3: 3,5,3-triiodothyronine
T4: Thyroxine
rT3: Reverse triiodothyronine
T2: Diiodothyronine
GAPDH: Glyceraldehyde 3-phosphate dehydrogenase
cKM: Creatine kinase muscle

## References

[1] Soleimani AF, Zulkifli I (2010). Effects of high ambient temperature on blood parameters in Red Jungle fowl, Village fowl and broiler chickens. J Anim Vet Adv.

[2] Ismail IB, Al-Busadah KA, El-Bahr SM (2013). Oxidative stress biomarkers and biochemical profile in broilers chicken fed zinc bacitracin and ascorbic acid under hot climate. Am J Biochem Mol Biol.

[3] Al-Zhgoul MB, Dalab AE, Ababneh MM, Jawasreh KI, Al Busadah KA, Ismail ZB (2013). Thermal manipulation during chicken embryogenesis results in enhanced Hsp70 gene expression and the acquisition of thermotolerance. Res Vet Sci.

[4] Moraes VMB, Malherios RD, Bruggman V, Collin A, Tona K, VanAs P (2003). Effect of thermal conditioning during embryonic development on aspects of physiological responses of broiler to heat stress. J Therm Biol.

[5] Piestun Y, Halevy O, Yahav S (2009). Thermal manipulations of broiler embryos--the effect on thermoregulation and development during embryogenesis. Poult Sci.

[6] Piestun Y, Harel M, Barak M, Yahav S, Halevy O (2009). Thermal manipulations in late-term chick embryos have immediate and longer term effects on myoblast proliferation and skeletal muscle hypertrophy. J Appl Physiol.

[7] Shinder D, Rusal M, Giloh M, Yahav S (2009). Effect of repetitive acute cold exposures during the last phase of broiler embryogenesis on cold resistance through the life span. Poult Sci.

[8] Walstra I, Ten Napel J, Kemp B, van den Brand H (2010). Temperature manipulation during layer chick embryogenesis. Poult Sci.

[9] Willemsen H, Kamers B, Dahlke F, Han H, Song Z, Ansari Pirsaraei Z (2010). High- and low-temperature manipulation during late incubation: effects on embryonic development, the hatching process, and metabolism in broilers. Poult Sci.

[10] Yahav S, Collin A, Shinder D, Picard M (2004). Thermal manipulations during broiler chick embryogenesis: effects of timing and temperature. Poult Sci.

[11] Yahav S, Rath R, Sasson A, Shinder D (2004). The effect of thermal manipulations during embryogenesis of broiler chicks (Gallus domesticus) on hatchability, body weight and thermoregulation after hatch. J Therm Biol.

[12] Lin CF, Asghar A, Gray JI, Buckley DJ, Booren AM, Crackel RL (1989). Effects of oxidised dietary oil and antioxidant supplementation on broiler growth and meat stability. Br Poult Sci.

[13] Melesse A, Maak S, Schmidt R, von Lengerken G (2011). Effect of long-term heat stress on key enzyme activities and T3 levels in commercial layer hens. Int J Livest Prod.

[14] Badran A, Desoky A, Abo-Eita E, Stino F (2012). Epigenetic thermal adaptation of chickens during late embryonic development. Egy Poult Sci.

[15] Loyau T, Me’tayer-Coustard S, Berri C, Crochet S, Cailleau-Audouin E, Crochet S (2014). Thermal manipulation during embryogenesis has long-term effects on muscle and liver metabolism in fast-growing chickens. PLoS One.

[16] McNabb FMA (1988). Peripheral thyroid hormone dynamics in precocial and altricial avian development. Am Zool.

[17] Arieli A, Berman A (1979). The effect of thyroxine on thermoregulation in the mature fowl (Gallus domesticus). J Therm Biol.

[18] Lam SK, Harvey S (1990). Thyroid regulation of body temperature in anaesthetized chickens. Comp Biochem Physiol A Comp Physiol.

[19] Yalčin S, Bruggeman V, Buyse J, Decuypere E, Çabuk M, Siegel PB (2009). Acclimation to heat during incubation: 4. Blood hormones and metabolites in broilers exposed to daily high temperatures. Poult Sci.

[20] Kalamah MAA (2001). Some physiological responses to heat stress in bronze turkey toms. Egypt Poult Sci.

[21] Al-Aqil A, Zulkifli I (2009). Changes in heat shock protein 70 expression and blood characteristics in transported broiler chickens as affected by housing and early age feed restriction. Poult Sci.

[22] Melesse A (2000). Comparative studies on performance and physiological responses of Ethiopian indigenous (Angete-Melata) chickens and their F1-crosses to long-term heat exposure.

[23] Melesse A, Maak S, Lengerken G, Pingel H (1998). Effect of longterm heat stress on the activity of blood enzymes and egg production traits in different chicken lines. Proceedings of 10th European Poultry Conference. June 21-26, Jerusalem, Israel.

[24] Ward MA, Peterson RA (1973). The effect of heat exposure on plasma uric acid, lactate dehydrogenase, chloride, total protein and zinc of the broiler. Poult Sci.

[25] Khone HJ, Jones JG (1975). Changes in plasma electrolyte acid-base balance and other physiological parameters of adult female turkeys under conditions of acute hyperthermia. Poult Sci.

[26] Ait-Boulahsen A, Garlich JD, Edens FW (1989). Effect of fasting and acute heat stress on body temperature, blood acidbase and electrolyte status in chickens. Comp Biochem Physiol A Comp Physiol.

[27] McDaniel CD, Bramwell RK, Howarth B (1996). The male contribution to broiler breeder heat-induced infertility as determined by sperm-egg penetration and sperm storage within the hen’s oviduct. Poult Sci.

[28] McDaniel CD, Bramwell RK, Wilson JL, Howarth B (1995). Fertility of male and female broiler breeders following exposure to elevated ambient temperatures. Poult Sci.

[29] Zulkifli I, Htin NN, Alimon AR, Loh TC, Hair-Bejo M (2007). Dietary selection of Fat by heat-stressed broiler chickens. Asian Australas J Anim Sci.

[30] Piestun Y, Shinder D, Ruzal M, Halevy O, Brake J, Yahav S (2008). Thermal manipulations during broiler embryogenesis: effect on the acquisition of thermotolerance. Poult Sci.

[31] Piestun Y, Shinder D, Ruzal M, Halevy O, Yahav S (2008). The effect of thermal manipulations during the development of the thyroid and adrenal axes on in-hatch and post-hatch thermoregulation. J Therm Biol.

[32] Tona K, Onagbesan O, Bruggeman V, Collin A, Berri C, Duclos MJ (2008). Effects of heat conditioning at d 16 to 18 of incubation or during early broiler rearing on embryo physiology, post-hatch growth performance and heat tolerance. Arch Geflugel.

[33] Bodine SC, Latres E, Baumhueter S, Lai VK, Nunez L, Clarke BA (2001). Identification of ubiquitin ligases required for skeletal muscle atrophy. Science.

[34] Gomes MD, Lecker SH, Jagoe RT, Navon A, Goldberg AL (2001). Atrogin-1, a muscle-specific F-box protein highly expressed during muscle atrophy. Proc Natl Acad Sci U S A.

[35] Dehoux M, Van Beneden R, Pasko N, Lause P, Verniers J, Underwood L (2004). Role of the insulin-like growth factor I decline in the induction of atrogin-1/MAFbx during fasting and diabetes. Endocrinology.

[36] Sandri M, Sandri C, Gilbert A, Skurk C, Calabria E, Picard A (2004). Foxo transcription factors induce the atrophy-related ubiquitin ligase atrogin-1 and cause skeletal muscle atrophy. Cell.

[37] Kamei Y, Miura S, Suzuki M, Kai Y, Mizukami J, Taniguchi T (2004). Skeletal muscle FOXO1 (FKHR) transgenic mice have less skeletal muscle mass, down-regulated Type I (slow twitch/red muscle) fiber genes, and impaired glycemic control. J Biol Chem.

[38] Stitt TN, Drujan D, Clarke BA, Panaro F, Timofeyva Y, Kline WO (2004). The IGF-1/PI3K/Akt pathway prevents expression of muscle atrophy-induced ubiquitin ligases by inhibiting FOXO transcription factors. Mol Cell.

